# Integrated use of chemical and geophysical monitoring to study the diesel oil biodegradation in microcosms with different operative conditions

**DOI:** 10.1007/s40201-021-00681-2

**Published:** 2021-05-28

**Authors:** Carla Maria Raffa, Andrea Vergnano, Fulvia Chiampo, Alberto Godio

**Affiliations:** 1 Department of Applied Science and Technology, Politecnico di Torino, Torino, Italy; 2 Department of Environment, Land and Infrastructure Engineering, Politecnico di Torino, Torino, Italy

**Keywords:** Aerobic bioremediation, Diesel oil removal, Fluorescein production, Water content, C/N ratio, Complex dielectric permittivity

## Abstract

This study aimed to monitor the aerobic bioremediation of diesel oil-contaminated soil by measuring: a) the CO_2_ production; 2) the fluorescein production; 3) the residual diesel oil concentration. Moreover, the complex dielectric permittivity was monitored through an open-ended coaxial cable. Several microcosms were prepared, changing the water content (u% = 8–15% by weight), the carbon to nitrogen ratio (C/N = 20–450), and the soil amount (200 and 800 g of dry soil). The cumulative CO_2_ and fluorescein production showed similar trends, but different values since these two parameters reflect different features of the biological process occurring within each microcosm. The diesel oil removal efficiency depended on the microcosm characteristics. After 84 days, in the microcosms with 200 g of dry soil, the highest removal efficiency was achieved with a water content of 8% by weight and C/N = 120, while in the microcosms with 800 g of dry soil the best result was achieved with the water content equal to 12% by weight and C/N = 100. In the tested soil, the bioremediation process is efficient if the water content is in the range 8–12% by weight, and C/N is in the range 100–180; under these operative conditions, the diesel oil removal efficiency was about 65–70% after 84 days. The dielectric permittivity was monitored in microcosms with 200 g of dry soil. The open-ended coaxial cable detected significant variations of both the real and the imaginary component of the dielectric permittivity during the bioremediation process, due to the physical and chemical changes that occurred within the microcosms.

## Introduction

The soil contamination by hydrocarbons is a current problem due to anthropic activities, that should be solved in an environmentally compatible way [1]. A good alternative to intensive chemical and physical operations is the bioremediation process. This approach is considered very effective because most of the hydrocarbons are biodegradable and many microbial species have been identified as degraders of these pollutants [2].

Different biological remediation approaches were studied and compared (i.e. natural attenuation, bioaugmentation, biostimulation) to evaluate the most efficient one for the degradation of a specific contaminant [3–5]. The choice of the technique depends on the type of pollutant, on the soil chemical and physical properties, and the environmental ones. Thus, before in-field application, laboratory experiments, initial field assessments, and pilot tests should be conducted to evaluate the biodegradability of hydrocarbon contaminants considering the soil conditions [6].

Focusing on the biostimulation, the growth of indigenous microorganisms is enhanced by optimizing physical and chemical process conditions to get efficient pollution degradation [7]. In several biostimulation studies, researchers have examined and monitored different parameters that influence the process: the water content [8], the pollutant concentration [9], the biomass concentration [10] or the quantity of nutrients [11].

Since each soil has its biological diversity and requirements, the optimal water content (u%) and the carbon to nitrogen ratio (C/N) are crucial in biostimulation process: 1) the water content allows the dispersion of soil aggregates and the contaminant diffusion in the aqueous phase, and promotes the contact between microorganisms and pollutants [12]; 2) the carbon to nitrogen ratio is useful to define the correct amount of nutrients since their excessive or scarce amount can inhibit the biological activity [13, 14].

Furthermore, a good indirect method to evaluate the biodegradation process is monitoring the changes in geophysical parameters due to pollutant removal and biological metabolism [15]. The geophysical methods to monitor degradation phenomena of hydrocarbon impacted sites are widely reported in several studies [16–19].

Geophysical methods based on the electrical and electromagnetic parameters are adopted both at laboratory scale [20] and in the field [21]. Researches focused on the integration of electrical conductivity, induced polarization, and self-potential measurements to detect subsurface contaminants [22]. A correlation between the self-potential signals and the in situ redox potentials was demonstrated both in the field and in the laboratory experiments [23]. The measured parameters are sensitive to the contaminant chemistry and the processes generated by bacteria during the biodegradation activity [24]. Electromagnetic monitoring of contaminated soil focused on the use of ground penetrating radar (GPR) in the assessment of hydrocarbon contaminations [25] and time domain reflectometry (TDR) to monitor the dielectric properties of soil–organic mixtures [26]. In this context, we focused on the detection of biodegradation related processes in contaminated soil by using an open-ended coaxial probe, because of its ability to provide a wide range of information about the dielectric properties of a material at different frequencies. The permittivity values are of interest in providing information on nature and saturation of the liquid phase of the soil matrix and in detecting chemical-physical phenomena related to the degradation activity.

Dielectric properties of soil mixtures with hydrocarbons have been observed using an open-ended coaxial probe, with a similar device adopted in the present study [27]. The open-ended coaxial probe is assimilated to the truncated section of a transmission line. The electromagnetic field propagates along the coaxial line and reflection occurs when the electromagnetic field encounters an impedance mismatch between the probe and the soil sample. The reflected amplitude is usually collected at different frequencies in a broad range and then converted into complex permittivity values. The optimal open-ended coaxial measurements are performed on a homogeneous sample and with good contact between the probe and the soil sample. Heterogeneities close to the surface could provide inaccurate measurements, particularly at a frequency lower than 1 GHz (heterogeneous samples could be particularly challenging). The theoretical limits of the measurement are generally estimated experimentally by calibrating the measure using liquids with different electrical permittivity values.

We prepared microcosms with different amounts of soil, water content, and carbon to nitrogen ratio (C/N). The contamination was simulated by adding commercial diesel oil. During the experimental runs, carbon dioxide and fluorescein production, and residual diesel oil were monitored and analyzed, to define the optimal water content and carbon to nitrogen ratio for the pollutant biodegradation. The soil complex dielectric permittivity was measured by an open-ended coaxial cable at the starting and the end of the test (84 days) on 200-g microcosms. Measurements were repeated to check the reliability of the approach in time-lapse monitoring of biodegradation phenomena.

## Materials and methods

The study was carried out on microcosms using sieved soil having the same origin and properties. Each microcosm consisted of a sealed glass jar filled with different amounts of soil, spiked with the same amount of diesel oil (70 g/kg of dry soil), and kept in different conditions of water content and C/N ratio.

In each microcosm, the clean-up process was monitored by: CO_2_ production, fluorescein production, residual diesel oil concentration. Besides, the complex dielectric permittivity of one series of microcosms was measured at the starting and the end of the test with an open-ended coaxial cable.

### Soil properties

The soil used in this work came from a non-contaminated area in Northern Italy, taken at a depth around 3 m under the surface. The soil granulometry was between 0.15 and 2 mm, sieved according to ASTM C method 136. Typical soil characteristics were measured in a previous study [28], namely: porosity = 40–42% by volume; grain density = 2700 kg/m^3^ and dielectric permittivity of dry soil = 2.5–3.

Table 1 reports the main properties of the tested soil, determined with standard methods.

**Table 1:** Soil parameters

Parameter	Value
pH	7.32 ± 0.04
Electrical conductivity (μS/cm)	165 ± 5
Bicarbonate (mg/kg)	66.9 ± 10.8
Ammonia (mg/kg)	2.18 ± 0.11
Nitrate (mg/kg)	68.0 ± 0.4
Chloride (mg/kg)	26.2 ± 0.3
Sulphate (mg/kg)	211 ± 3

The elemental characterization of the tested soil is reported in Table 2. The Inductively Coupled Plasma Mass Spectrometry (ICP-MS) was used to detect these elements.

**Table 2:** Elemental characterization of soil samples with different granulometry

Chemical element	Concentration (% by weight)		
	1 (0.15–0.5 mm)	2 (0.5–1 mm)	3 (1–2 mm)
Si	18.107	20.534	4.260
Ca	15.013	11.022	20.443
Al	6.339	6.679	5.966
Fe	5.552	5.817	3.113
B	3.939	3.032	3.190
K	1.636	1.615	1.535
Na	1.267	1.030	1.439
Mn	0.397	0.319	0.364
Mg	0.285	0.297	1.116
Zn	0.090	0.078	0.080
V	0.066	0.063	0.071
Cr	0.057	0.079	0.164
Sr	0.043	0.046	0.077
Sb	0.042	0.038	0.052
Ni	0.036	0.040	0.052
Cu	0.033	0.035	0.035
Ba	0.012	0.009	0.018
Co	0.010	0.009	0.011
As	0.008	0.006	0.007
Pb	0.006	0.006	0.007
Cd	0.002	0.002	0.003
Be	0.002	0.002	0.002
Mo	0.002	0.001	0.001
Se	*	*	*
Tl	*	*	*
Ag	*	*	*
Hg	*	*	*

*Concentration < 0.001% by weight

### Soil microcosms

Two sets of microcosms were realized with different amounts of soil: 200 g and 800 g. Microcosms were set up in sealed glass jars:

- For microcosms with 200 g of soil: jar volume = 0.2 l, jar diameter = 7 cm and soil layer height = 3 cm;
- For microcosms with 800 g of soil: jar volume = 1 l, jar diameter = 9.5 cm and soil layer height = 6 cm.

The systems were hydrated with Mineral Salt Medium for Bacteria (MSMB) [29] to enhance the microbial activity of indigenous bacteria and aerated 2–3 times a week by manual mixing with a laboratory spoon for 5 min to promote the aerobic process.

The water content (u%) and the carbon to nitrogen ratio (C/N) were changed dosing the MSMB solution to obtain:

- For microcosms with 200 g of dry soil: u% = 8%, 12% and 15% by weight and C/N = 60, 120, 180 and 300 (12 microcosms in total);
- For microcosms with 800 g of dry soil: u% = 12% by weight and C/N = 20, 100, and 450 (3 microcosms in total).

The carbon to nitrogen ratio took into account also the carbon and nitrogen content of the soil.

Each microcosm was artificially contaminated with a commercial diesel oil at a concentration equal to 70 g/kg of dry soil. This pollution concentration is the limit to avoid saturation of the tested soil by the diesel oil.

Preliminary experimental tests were carried out in the microcosms with 200 g of dry soil, for 35 days. Then, on the base of the results, the microcosms with 800 g of dry soil were set-up and monitored for 84 days. About microcosms with 200 g of dry soil, the ones showing the highest diesel oil removal efficiency were monitored until the 84th day, too.

### Respirometric measurements

The CO_2_ production was measured by acid-base titration along the tests, measuring the CO_2_ absorption in a 1.5 M NaOH solution, as by the reaction:

1 $$ {\mathrm{CO}}_2+2\mathrm{NaOH}\to {\mathrm{Na}}_2{\mathrm{CO}}_3+{\mathrm{H}}_2\mathrm{O} $$

In each microcosm, 20 ml of NaOH solution was put in a cylindrical container to promote the CO_2_ absorption. Every 2–3 days, the NaOH solution was manually titrated with 1.5 M HCl, and the phenolphthalein was used as an indicator. After each titration, the NaOH solution was replaced.

The produced CO_2_ was determined considering the amount of HCl used in the titration.

Considering the mass balance, the CO_2_ amount was obtained as:

2 $$ {\mathrm{m}}_{\mathrm{CO}2}=1/2\cdot {\mathrm{M}\mathrm{M}}_{\mathrm{CO}2}\cdot \left({\mathrm{V}}_{\mathrm{HCl},0}-{\mathrm{V}}_{\mathrm{HCl},1}\right)\cdot {\mathrm{M}}_{\mathrm{HCl}} $$

where,

m_CO2_: is the produced CO_2_ amount (mg);
MM_CO2_: is the CO_2_ molar mass (g/mol);
M_HCl_: is the HCl molarity (mol/l);
V_HCl,0_: is the initial HCl volume (ml);
V_HCl,1_: is the final HCl volume (ml).

The CO_2_ amount produced along the tests reflects the respiratory activity of the microorganisms in the microcosm.

### Fluorescein diacetate (FDA) analysis

The microbial activity was evaluated using the simple and fast method of hydrolysis of fluorescein diacetate (FDA) [30–32].

FDA is hydrolyzed by enzymes active in biodegradation processes (protease, lipase, esterase) with fluorescein as the final product. The produced fluorescein amount is determined by spectrophotometric analysis.

To promote FDA hydrolysis, two solutions were prepared:

- Potassium phosphate buffer: aqueous solution with 8.7 g/l of K_2_HPO_4_ and 1.3 g/l of KH_2_PO_4_ at pH = 7.6;
- FDA stock solution in acetone: 0.1 g of FDA in 50 ml of acetone.

From each microcosm, 2 g of wet soil was sampled and was mixed into 15 ml of potassium phosphate buffer and 100 μl of FDA stock solution in acetone. The solution was agitated at 50 rpm for 1 h. After sample centrifugation at 6000 rpm for 5 min, 15 ml of acetone was added to stop the hydrolysis reaction. The liquid was filtered through a 1.2 μm filter to remove possible colloidal particles. The solution absorbance was measured via spectrophotometric analysis at 490 nm, with respect to the blank that contained only the potassium phosphate buffer.

The analysis was done 2–3 times a week, and for a better understanding of the microbial process, two different periods were monitored, namely: at t = 0–35 days for microcosms with 200 g of dry soil and at t = 20–84 days for the ones with 800 g of dry soil. For each microcosm, two replicates were done.

### Residual diesel oil concentration

In each microcosm, the extraction of hydrocarbons was done according to the EPA method 3546 (moisture 15% - 30% by weight), based on microwave heating.

A sample of 2 g of wet soil was taken from each microcosm and mixed with 30 ml of solvent (acetone and *n*-hexane with 1:1 by volume) and 2 g of anhydrous sodium sulfate. Two replicates were done for each sample.

The samples were put in a microwave oven, where the thermal cycle was:

- Heating at 110 °C and power 1100 W for 10 min;
- Constant temperature at 110 °C and power 1100 W for 10 min;
- Cooling for 20 min.

When the extraction was concluded, the sample was filtrated through a 0.45 μm filter.

The residual diesel oil was measured according to the EPA method 8015. The extracted samples were analyzed using a gas chromatograph equipped with flame ionization detector and DB-5 fused silica capillary column, operated with helium as a carrier, and with injector and detector maintained at 220 °C and 250 °C, respectively. This method defines the thermal cycle:

- Keeping at 50 °C for 1 min;
- Heating at 320 °C by rate 8 °C/min;
- Keeping at 320 °C for 40 min;
- Cooling at 50 °C.

The diesel oil concentration was determined using a calibration line done with the commercial diesel oil used in the tests. For each extract, the analysis was replicated twice.

The residual diesel oil concentration was monitored for 35 days for microcosms with 200 g of soil, whereas in microcosms with 800 g of soil it was measured just at t = 84 days. To compare the removal efficiency of the microcosms with different soil amounts on long times, the tests on 200-grams microcosms with the best diesel oil removal efficiency (C/N = 120 and C/N = 180) were prolonged until t = 84 days to measure the pollutant concentration at that time.

The diesel oil removal efficiency, η, was calculated as:

3 $$ \upeta =\left({\mathrm{C}}_0-{\mathrm{C}}_1\right)/{\mathrm{C}}_0\cdot 100 $$

where C_0_ = 70 g/kg of dry soil is the initial diesel oil concentration and C_1_ is the final one.

### Complex dielectric permittivity with open-ended coaxial cable

We chose to use an open-ended coaxial probe since it measures both the real part and the imaginary part of the complex dielectric permittivity, which reflect different properties of the soil matrix. The changes of the real part are closely related to changes in the water content, while changes in the imaginary part are mainly linked to chemical variations in the pore water that affects the electrical conductivity.

The dielectric permittivity (ε) is a measure of the polarization of a material when it is located in an electric field. The higher its dielectric permittivity, the greater the alignment of its molecules to the electric field. Often, it is normalized to the dielectric permittivity of vacuum (ε_0_) and is called relative permittivity or dielectric constant (ε_r_).

4 $$ {\upvarepsilon}_{\mathrm{r}}=\upvarepsilon /{\upvarepsilon}_0 $$

This dimensionless property has peculiar values for each material (e.g. for water = 80, for air = 1), so that its measurement can be used to identify different materials. In particular, in multiphase materials such as soil, the dielectric constant allows to evaluate the relative quantity of each phase, e.g. the water content. A simple correlation between dielectric constant and water content has been calculated by using Topp’s model and CRIM model [33].

The dielectric permittivity, however, is a complex quantity in which the real and the imaginary part have different physical meanings: the first represents the strength of the electric field created by the polarization of the material, the latter the dielectric and conductive losses. Besides, the dielectric permittivity is frequency dependent, because different polarization mechanisms happen at different frequency ranges. Therefore, the analysis of a frequency spectrum of both real and imaginary components of dielectric permittivity is more complete information about the dielectric properties of a material, compared to a single value of dielectric constant. This is particularly true if a complex multiphase matrix is studied, such as contaminated soil, which is a 4-phase material (soil grains, air, water, contaminant). Our needs are also to evaluate the evolution of dielectric properties of that material during a biological process of degradation.

The experimental setting consisted of an open-ended coaxial probe connected to a network analyzer capable of measuring the complex dielectric permittivity in a frequency range from 0.2 GHz to 20 GHz. The specimens under test were previously homogenized by mechanical mixing. The measures were performed by keeping in contact the probe with the soil; the probe was connected to the network analyzer, which acquires the spectral response of the dielectric permittivity.

The measurements of the complex dielectric permittivity were performed in the microcosms with 200 g of dry soil and C/N = 120 and C/N = 180, the at t = 0 and t = 84 days; the main aim was to confirm that the real and imaginary parts of the dielectric permittivity are linked to the diesel oil biodegradation. Moreover, they provide useful indirect information about water and contaminant content, and other parameters, such as salinity, in the microcosms [15].

For each microcosm, we repeated ten separated observations of the complex dielectric permittivity spectrum to estimate the standard deviation of the measures: one of the goals was evaluating if this technology can provide reliable and reproducible results in the bioremediation context.

## Results

### Respirometric measurements

#### Microcosms with 200 g of dry soil

**Fig. 1:**
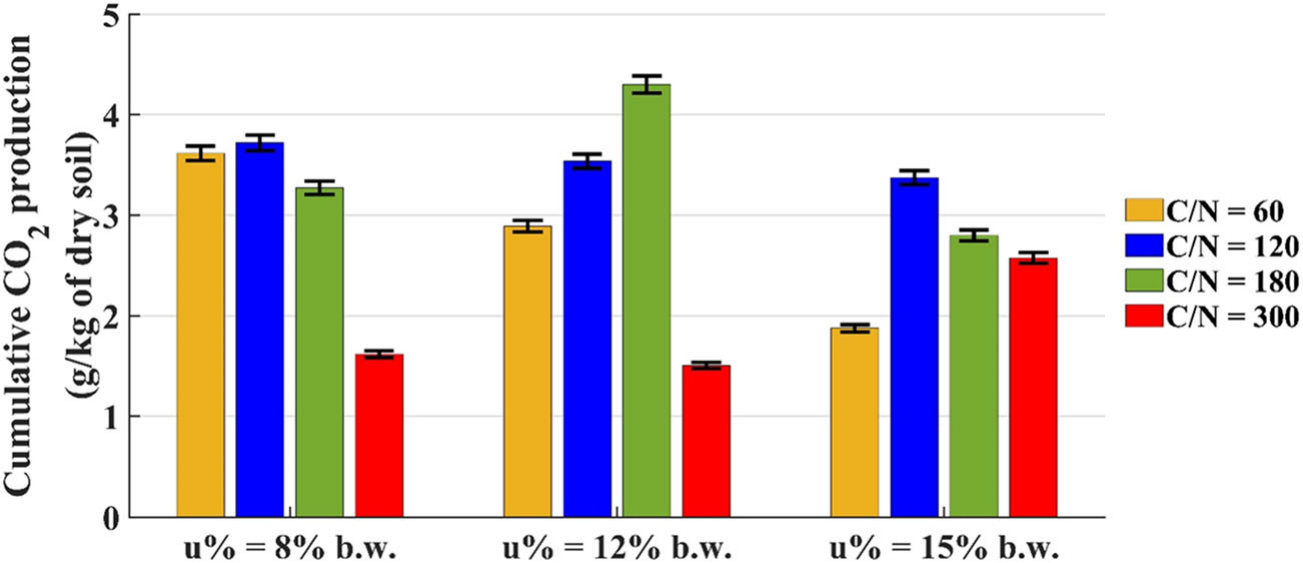
Cumulative CO_2_ production by microcosms with 200 g of dry soil at t = 30 days

The influence of water content (u%) and carbon to nitrogen ratio (C/N) over the cumulative CO_2_ production at t = 30 days is shown in Fig. 1.

In general, the microcosms with C/N = 120 and C/N = 180 produced a higher CO_2_ amount than the others. The highest CO_2_ production was 4.3 g/kg of dry soil (u% = 12% b.w. and C/N = 180), followed by 3.7 g/kg of dry soil (u% = 8% b.w. and C/N = 120). Figure 1 shows that the nutrient concentration influences the respiratory activity; in particular, in the systems with extreme values (C/N = 60 and C/N = 300), the degradative process is penalized due to excessive or insufficient amount of nitrogen required to the microbial growth. The CO_2_ production is influenced less by the water content than by the C/N ratio.

**Fig. 2:**
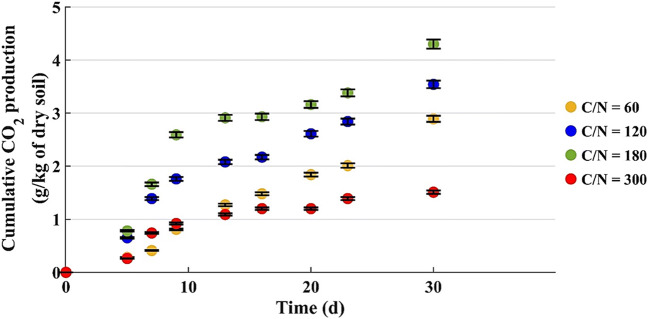
Cumulative CO_2_ production by microcosms with 200 g of dry soil (u% = 12% b.w.)

Figure 2 shows the monitoring for the microcosms with u% = 12% by weight. In the microcosms with C/N = 60 and 300, the CO_2_ production was the same in the first 10 days; then, the microcosm with C/N = 60 started to increase at the rate exhibited by the ones with C/N = 120 and 180, whereas the production for C/N = 300 grew very slowly.

#### Microcosms with 800 g of dry soil

The results of microcosms with 200 g of dry soil showed that the best condition was achieved with u% = 12% by weight. Based on these results, microcosms with 800 g of dry soil were set up with this water content and changing only the C/N ratio.

**Fig. 3:**
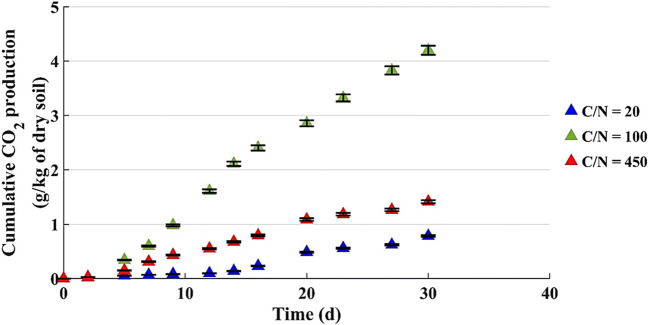
Cumulative CO_2_ production by microcosms with 800 g of dry soil (u% = 12% b.w.)

Figure 3 shows the CO_2_ production during the first 30 days of the test. The cumulative CO_2_ amounts are in line with the quantities achieved with 200 g of dry soil (Fig. 2).

**Fig. 4:**
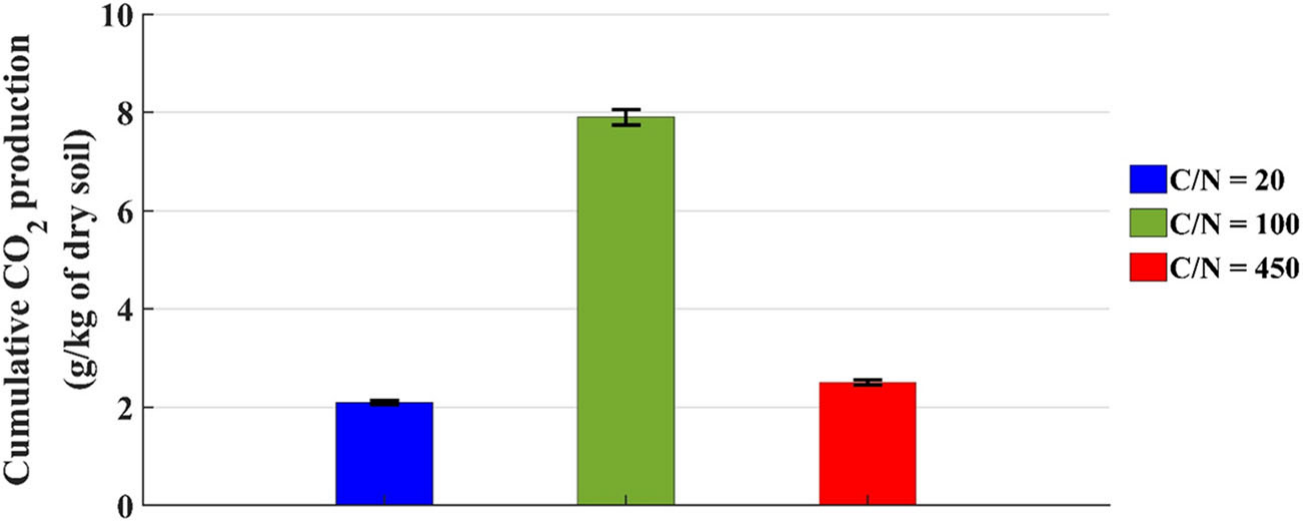
Cumulative CO_2_ production by microcosms with 800 g of dry soil at t = 80 days

In these microcosms, the CO_2_ was monitored for 80 days and the cumulative CO_2_ production at the end of the tests is reported in Fig. 4.

Considering Figs. 3 and 4, it can be noted that:

- In the first 5 days the respiratory activity was not evident;
- CO_2_ production trend is linear and its rate, to say the line slope, is higher for the microcosm with C/N = 100;
- The data of microcosms with C/N = 20 and 450 have the same trend, but the CO_2_ production started later in the microcosm with more nutrients (C/N = 20) than one in the microcosm with low nitrogen concentration (C/N = 450);
- In one month, the curves do not reach constant values: this is evidenced by the results achieved at t = 80 days and shown in Fig. 4, where the CO_2_ amount in each tested microcosm is almost double compared to the one measured at t = 30 days;
- The microcosm with C/N = 100 produced the highest amount of CO_2_, about 8 g/kg of dry soil after 80 days;
- For the microcosms with C/N = 20 and C/N = 450, the results at t = 80 days are similar: 2.1 and 2.5 g/kg of dry soil, respectively, and much lower than that of the system with C/N = 100. However, these results are completely in line with the ones for the biotic control achieved in a previous study [34] after 80 days; in particular, 2.1 g/kg of dry soil with C/N = 20 is equal to the result achieved in that study where the soil amount was different but the layer height was about the same (7 cm). This suggests the hypothesis that with C/N = 20 the microbial population is largely inhibited, and the respiratory activity is limited to the basal one, notwithstanding the presence of degradable substrate.

The CO_2_ results of microcosms with 200 and 800 g of soil and water content equal to 12% by weight confirm that for this biostimulation process the proper C/N ratio is in the range 100–180.

### Fluorescein diacetate (FDA) analysis

In general, the trend of cumulative fluorescein production agreed with those of CO_2_ production since both the parameters refer to the biological process occurring in the microcosm, as a whole. The FDA analysis has the advantage to measure a parameter linked to the microbial activity for pollution degradation and can be used as an indicator of the substrate transformation.

#### Microcosms with 200 g of dry soil

**Fig. 5:**
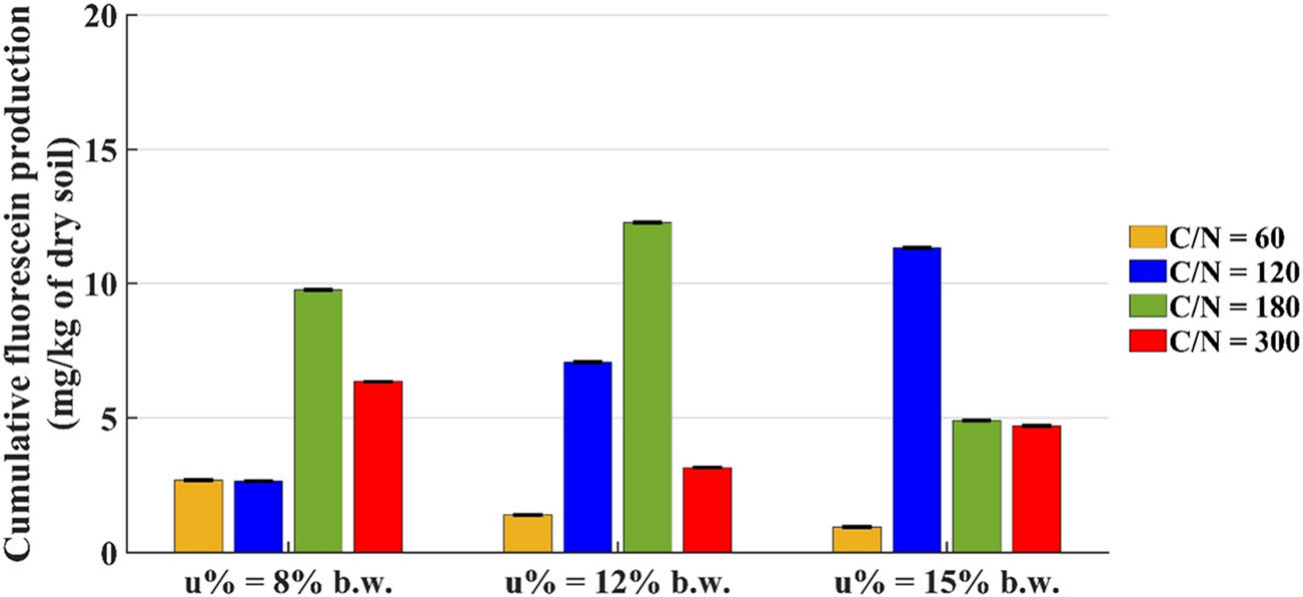
Cumulative fluorescein production by microcosms with 200 g of dry soil at t = 30 days

Figure 5 shows the fluorescein amount produced in 30 days in the microcosms with 200 g of dry soil.

The results show that the highest fluorescein production (12.3 mg/kg of dry soil) is found in the microcosm with u% = 12% b.w. and C/N = 180, to say the same microcosm that had the highest CO_2_ production. The other microcosm that produced much fluorescein is that with u% = 15% b.w. and C/N = 120, even if this is not completely in agreement with CO_2_ results. It must be said that the FDA hydrolysis method allows an overall estimate of microbial activity and not limited to the aerobic microorganisms used in the degradative process [30].

The systems with C/N = 180 are suitable for microbial activity with all water content conditions, except for u% = 15% by weight. Considering u% = 15% b.w., the microbial activity is more intense in the microcosm with C/N = 120 (fluorescein production is 11.3 mg/kg of dry soil).

**Fig. 6:**
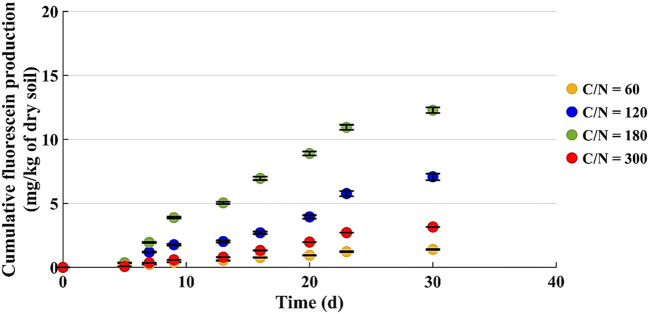
Cumulative fluorescein production by microcosms with 200 g of dry soil (u% = 12% b.w.)

Figure 6 shows the monitoring of the fluorescein production in the microcosms with u% = 12% by weight.

The curves increase almost linearly after the 5th day, then they change slope between the 23rd and 30th day, because the daily fluorescein production decreases.

#### Microcosms with 800 g of dry soil

In these tests, the microbial activity monitoring started at t = 20 days, to say 20 days from the beginning of the test and it was measured for 60 days, till t = 80 days, except for C/N = 100 (in this case monitoring started at t = 12 days).

**Fig. 7:**
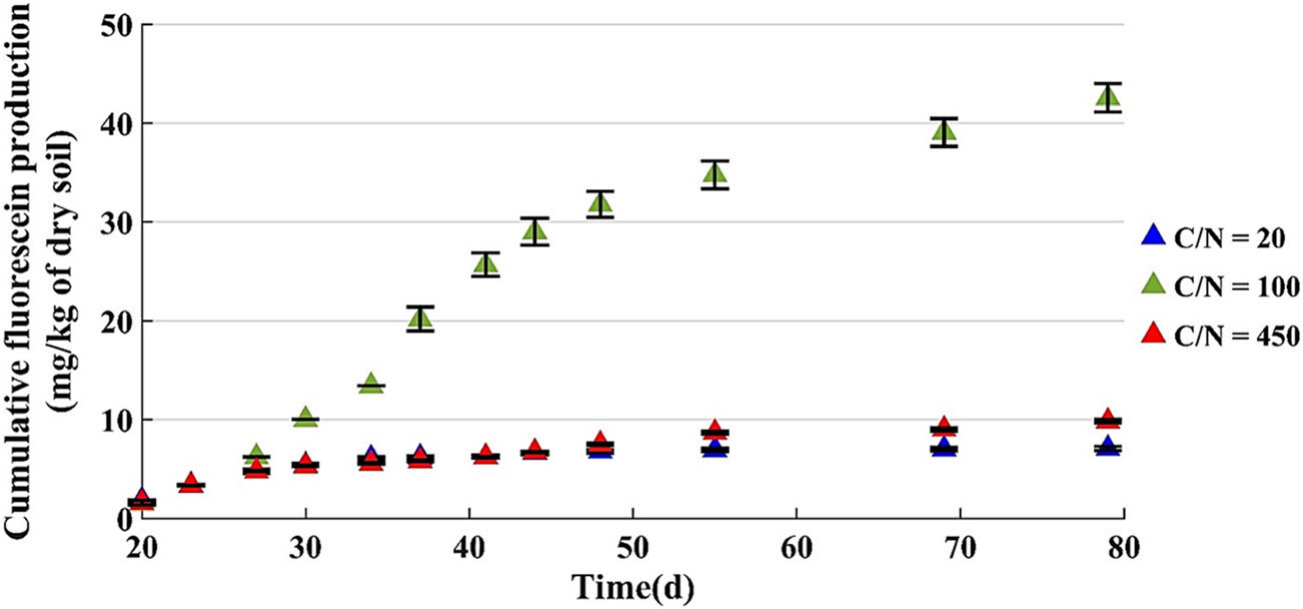
Cumulative fluorescein production by microcosms with 800 g of dry soil (u% = 12% b.w.)

The cumulative fluorescein production is reported in Fig. 7, where it can be seen that with C/N = 100 the amount is about 42.5 mg/kg of dry soil, while it is 7.2 and 9.8 mg/kg of dry soil with C/N = 20 and 450, respectively.

The curves have a linear trend between the 20th and 30th day for microcosms with C/N = 20 and 450, and between the 20th and 50th day for microcosm with C/N = 100; after this time, the cumulative fluorescein value grows slightly.

It can be noticed the different trends of the cumulative fluorescein production in the microcosms with u% = 12% b.w. and different amounts of soil (Figs. 6 and 7). The reason is probably the delayed measurement of fluorescein production in the microcosms with 800 g of dry soil.

Table 3 compares fluorescein production in the microcosms with water content equal to 12% by weight.

**Table 3:** Comparison of cumulative fluorescein production (mg/kg of dry soil) in the tested microcosms (u% = 12% b.w.)

Microcosms	C/N	20 days	23 days	30 days
200 g of dry soil	60	0.9	1.2	1.4
	120	3.9	5.8	7.1
	180	8.9	10.9	12.3
	300	2.0	2.7	3.2
800 g of dry soil	20	1.9	3.4	5.3
	100	1.6	3.3	10.0
	450	1.6	3.3	5.3

Between the 23rd and 30th day, the microbial activity was more intensive in the microcosms with 800 g of dry soil: for example, the produced fluorescein of the microcosm with C/N = 100 increased from 3.3 to 10.0 mg/kg of dry soil, to say by 6.7 mg/kg of dry soil, while in the microcosm with C/N = 180, in the same time, the cumulative fluorescein increased by 1.4 mg/kg of dry soil.

We tried to link the results of CO_2_ and fluorescein production, to highlight that these parameters are indicative of the whole microbial process.

**Fig. 8:**
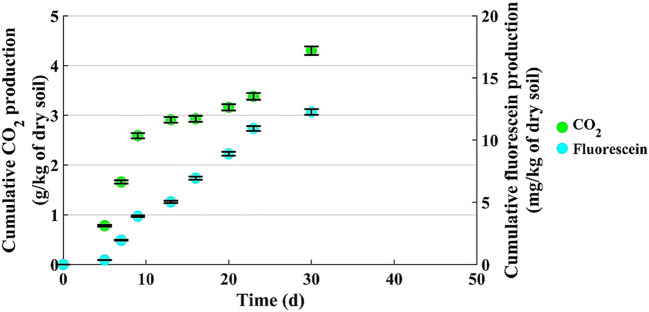
CO_2_ and fluorescein production by 200-grams microcosm (u% = 12% b.w. and C/N = 180)

**Fig. 9:**
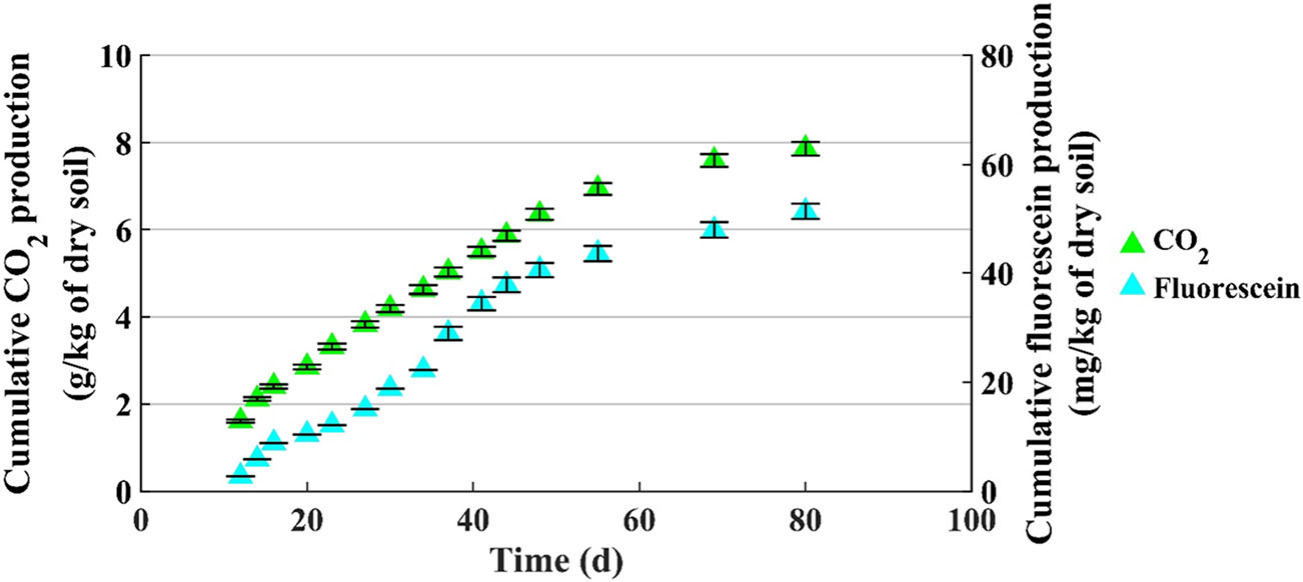
CO_2_ and fluorescein productions by 800-grams microcosm (u% = 12% b.w. and C/N = 100)

Figures 8 and 9 show the CO_2_ and fluorescein production for microcosm with 200 g of dry soil (u% = 12% b.w. and C/N = 180) and with 800 g of dry soil (u% = 12% b.w. and C/N = 100), respectively.

Figures 8 and 9 evidence some common features:

- The productions of CO_2_ and fluorescein do not start immediately with the test, but after acclimatization time, that can be estimated around 5 days;
- After the first period of linear growth, in both microcosms, the curves change slope, to say the production rate decreases, and this is more evident for the CO_2_ production.

To this purpose, to better highlight the process trend, the daily production of CO_2_ and fluorescein were calculated and shown in Table 4.

**Table 4:** Daily CO_2_ and fluorescein production

Microcosm	Daily CO_2_ production (g/d/kg of dry soil)		Daily fluorescein production (mg/d/kg of dry soil)	
	t = 5–10 days	t = 10–23 days	t = 5–23 days	t = 23–30 days
200 g of dry soil (C/N = 180)	0.45	0.056	0.59	0.19
	t = 12–55 days	t = 55–80 days	t = 12–55 days	t = 55–80 days
800 g of dry soil (C/N = 100)	0.10	0.029	0.95	0.31

These two parameters reflect different features of the biological process occurring within each microcosm. However, CO_2_ production keeps into account also the basal respiration, to say it can contain contributions not just due to the pollutant removal. Similar results are found in a study that evaluated the effect of soil amendment on the hydrolysis of the FDA and its relationship with microbial biomass and CO_2_ evolution [35].

The results for daily fluorescein production show a decrease along the time in both the microcosms, even if with different values. However, in both cases the decrease has the same entity: for 200-grams microcosm, 0.19/0.59 = 0.32, for 800-grams microcosm, 0.31/0.95 = 0.32.

### Residual diesel oil concentration

The diesel oil removal efficiency was measured at the end of the test, namely after 35 days for microcosms of 200 g of dry soil, except for microcosms with C/N = 120 and 180 that were analyzed also at t = 84 days. The diesel oil concentration of microcosms with 800 g of dry soil was evaluated at t = 84 days.

#### Microcosms with 200 g of dry soil

**Fig. 10:**
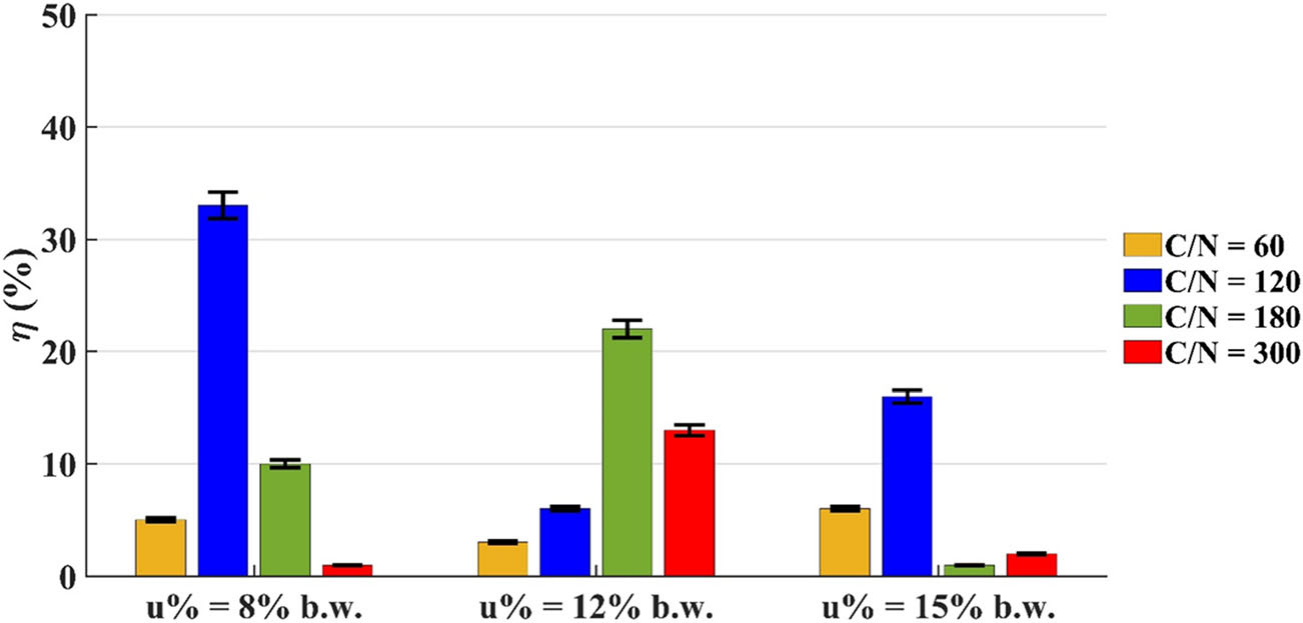
Diesel oil removal efficiency by microcosms with 200 g of dry soil at t = 35 days

The microcosm where the bioremediation process was the most efficient was the one with C/N = 120 and u% = 8% b.w. In this system, the initial diesel oil concentration decreased by 33%, as shown in Fig. 10. For microcosm with C/N = 60, the percentage of removed diesel oil was around 5% for the tested water contents. For systems with C/*N* = 300, the highest value was achieved with a water content equal to 12% b.w. (η = 13%), while in the other cases, it can be affirmed that the pollutant was not removed.

Considering that in 35 days the best removal efficiency was achieved with C/N = 120 (η = 33%) and C/N = 180 (η = 22%), we decided to extend the monitoring of the residual diesel oil concentration in these systems until t = 84 days.

Table 5 reports the results achieved in the two lines of microcosms.

**Table 5:** Diesel oil removal efficiency by microcosms with 200 g of dry soil at t = 84 days

	u% (by weight)		
	8%	12%	15%
C/N = 120	71%	60%	57%
C/N = 180	54%	60%	59%

#### Microcosms with 800 g of dry soil

**Fig. 11:**
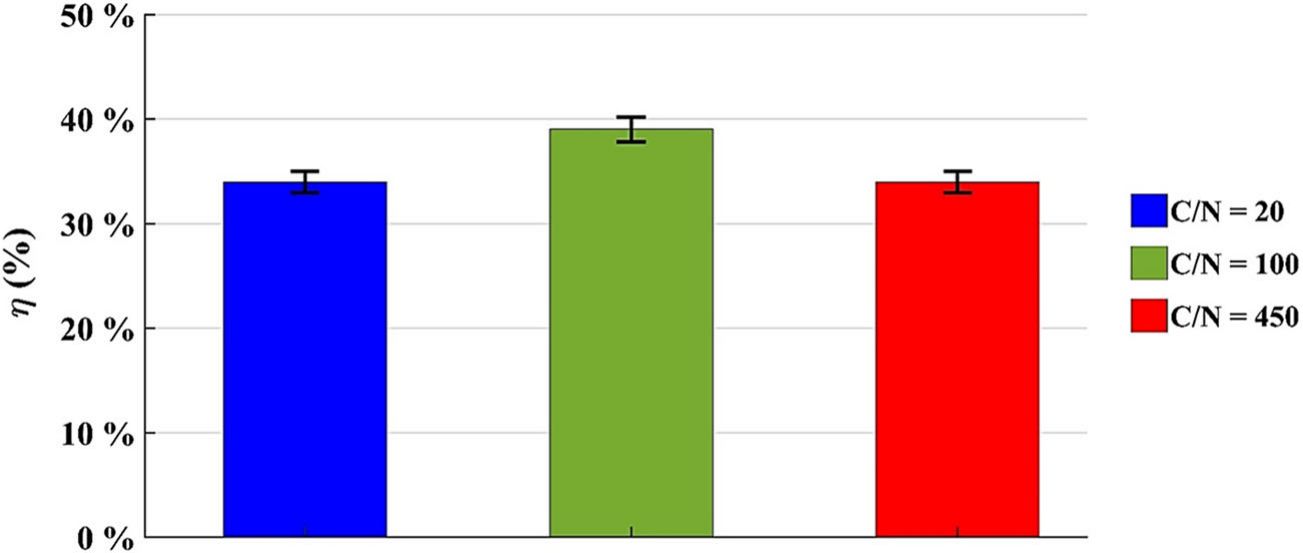
Diesel oil removal efficiency by microcosms with 800 g of dry soil at t = 84 days

In the microcosms with 800 g of soil the diesel oil concentration was determined just at t = 84 days and the diesel oil removal efficiency is reported in Fig. 11.

The data show that the diesel oil concentration moderately decreased in 84 days, even if the respirometric analysis and the FDA analysis suggested a better result.

The highest removal efficiency is about 39% with C/N = 100. Compared to the other results, this is the system where the bioremediation process is more efficient, even if the difference with the others is not so relevant since both have η = 34%. Similar removal efficiencies for total petroleum hydrocarbons in microcosms with 1000 g of soil were obtained by other Authors [36].

Looking at the results achieved with 200-grams microcosms, we observed a higher removal of diesel oil amount than the ones with 800 g of dry soil. In the system with C/N = 120 and u% = 12% b.w., the removal efficiency was about 60% of the initial concentration. In the worst case, namely C/N = 180 and u% = 8% b.w., η = 54% but higher than the ones (39% and 34%) with microcosms with 800 g of dry soil. This could be due to the greater amount of soil, which does not favor the oxygen diffusion and therefore the degradation process, despite the intense microbial activity.

### Open-ended coaxial cable measurements

**Fig. 12:**
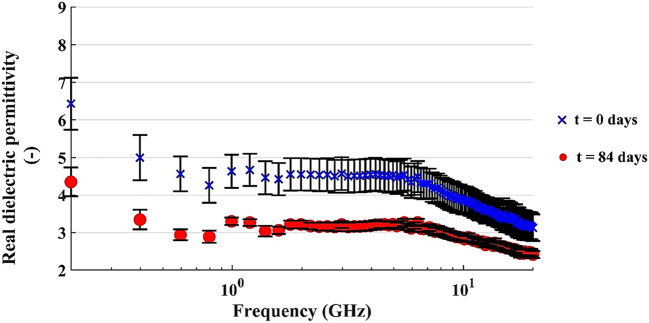
Real dielectric permittivity of the microcosm with 200 g of dry soil (C/N = 120 and u% = 12% b.w.); vertical bar refers to the standard deviation of the amplitude at each frequency

**Fig. 13:**
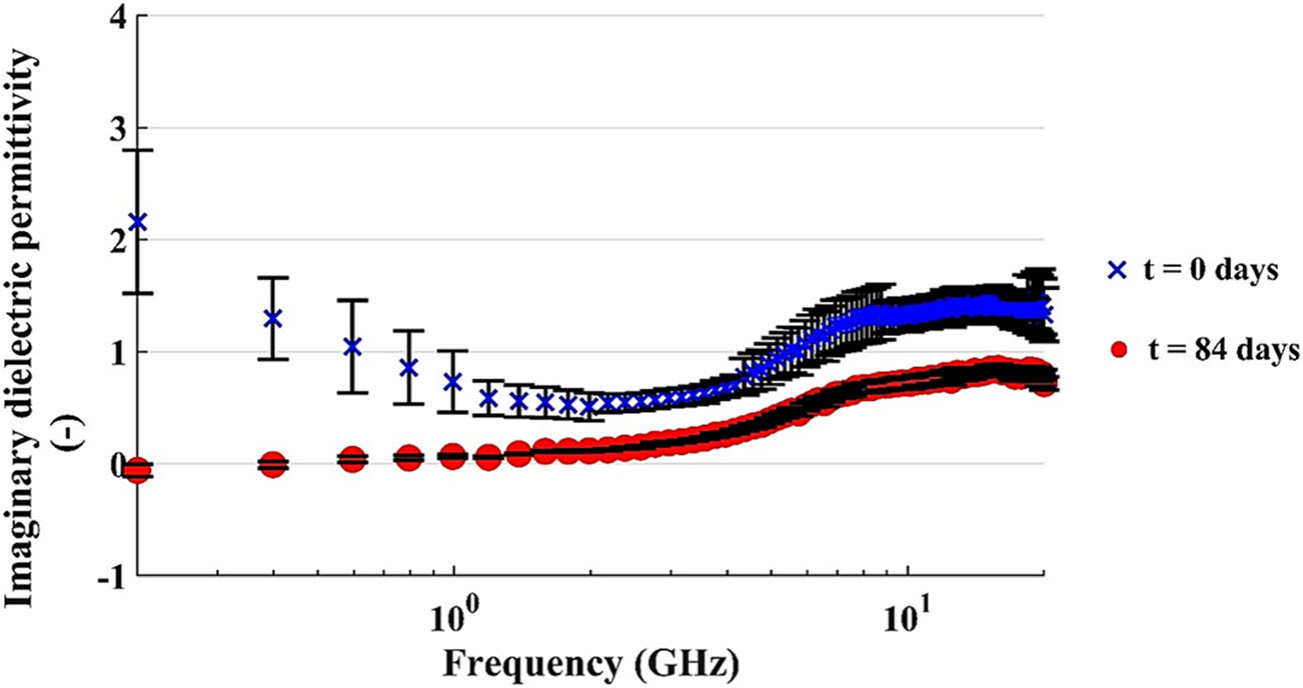
Imaginary dielectric permittivity of the microcosm with 200 g of dry soil (C/N = 120 and u% = 12% b.w.); vertical bar refers to the standard deviation of the amplitude at each frequency

The real and imaginary parts of the dielectric permittivity versus the frequency of the applied signal are reported in Figs. 12 and 13. The data are related to the microcosm with C/N = 120 and u% = 12% by weight. Similar trends were obtained for the other microcosms.

The plots of Fig. 12 compare the real part of dielectric permittivity before and after 84 days of bioremediation. The peculiar dependence of the permittivity on the frequency is related to the resonant frequency of the frequency spectrum. This is usually related to the solid-fluid interaction of porous materials. The absolute values lowered by 1 to 2 units, during the time of the experiment.

Figure 13 shows the trend of the imaginary part observed at the two reference times (0 and 84 days); the most interesting observation is that at frequencies lower than 1 GHz, where the imaginary part drastically decreases to values near zero, following a different evolution pattern compared to that of the real part, after 84 days.

## Discussion

In this study, the carbon dioxide, fluorescein production, and residual diesel oil concentration were measured along with the tests to evaluate the optimal condition for the biodegradation process.

Carbon dioxide and fluorescein production are parameters that reflect different features of the biodegradation process, namely respiratory activity (CO_2_) and microbial activity (fluorescein). The carbon dioxide production is often monitored to evidence the presence of microbial respiration, as demonstrated in previous studies [9, 10, 37].

The results achieved in our study confirmed that the microcosms with water content around 12% by weight and carbon to nitrogen ratio equal to 120 and 180 produced the highest amounts of CO_2_, as found also by Komilis et al. [38]. These researchers estimated the optimal C/N ratio in the range 40–100 in microcosms polluted with 2% by weight of diesel oil. However, this parameter takes also into account the basal respiration, to say an amount of CO_2_ not due to the pollutant degradation, and this amount could be not negligible, as shown by other studies [34, 35, 39]. In other words, the monitoring of CO_2_ production is not sufficient to get precise information on the biodegradation trend.

For this, fluorescein production was monitored in parallel to CO_2_ production, and the results showed that their trend was similar in all the tested microcosms.

The results achieved for both these parameters showed that the biodegradation process was never evident in the first 5 days or more. This delay is completely in line with studies by other researchers [39, 40]: the reason can be ascribed to the number of complex molecules (diesel oil compounds) that are not easily and quickly degradable by indigenous microorganisms. Vice versa, with simple compounds, their fast removal is shown by the rate of respiration activity, to say the CO_2_ production rate [35].

About the diesel oil removal, the best results were achieved for microcosms with C/N around 120, whichever the water content. Our results and those found by other authors [10, 37] suggest that the removal efficiency is influenced less by the water content than by the C/N ratio. In particular, our findings support the idea of process inhibition by nitrogen content, more than its depletion, when the microbial activity could continue in the endogenous respiration phase.

The test duration showed a relevant influence, as can be noted comparing the diesel oil removal efficiency in 200-grams microcosms at t = 35 days to the one at t = 84 days. About the microcosms with 800 g of dry soil, after 84 days the removal efficiency was 39% with C/N = 100 and around 34% with C/N = 20 and C/N = 450, to say not much lower. Similar removal efficiencies for total petroleum hydrocarbons in microcosms with 1000 g of dry soil were obtained by Fan et al. [36].

These values are rather lower than the ones achieved for smaller microcosms (200 g of dry soil) at t = 84 days: the reason could be the amount of soil, that can limit the oxygen diffusion in some parts of the microcosm, notwithstanding the soil aeration every 3–4 days. As already highlighted in a previous study [41], the diesel biodegradation rates determined with small-scale experiments (30 g and 60 g of soil) are not sufficiently representative of microbial variety, soil heterogeneity and oxygen distribution in the soil, therefore when they are compared with larger systems (0.4, 2 and 13 kg of soil), the efficiency of biodegradation is reduced.

The validity of the experimental activity and the derived results is inferred by the relative standard deviation, to say the ratio between the standard deviation and the measurement mean. Table 6 shows the range of this value for the parameters monitored in all the tested microcosms. As it can be noted, the ranges are rather narrow, supporting a good quality of the experimental runs and measurements.

**Table 6:** Relative standard deviation (%) range for CO_2_ and fluorescein production, and diesel oil removal efficiency (η) measurements in the tested microcosms

Parameter	Microcosms	
	200 g of dry soil	800 g of dry soil
CO_2_ production	0.01–2.83	0.02–2.83
Fluorescein production	0.02–2.04	0.02–5.92
η	2.34–2.53	1.20–1.41

The geophysical monitoring of the tests pointed out a decrease of the real component of the dielectric permittivity at all investigated frequencies from t = 0 to t = 84 days. This may be related to the loss of diesel compounds through biodegradation, as can be seen in a previous study [42]. However, the decrease in permittivity is too pronounced to reflect only the loss of diesel compounds, according to the CRIM model [33], so a slight loss in water content must be supposed, and probably it happened during the mixing operations. On the other hand, the imaginary part depends on the electrical conductivity of the microcosms, mainly at low frequencies. We observed a decrease particularly evident in the low-frequency range, between 0.2 and 2 GHz: we supposed that the nutrient salts, responsible for a decrease of the bulk electrical conductivity of the microcosms, were depleted by microbial metabolism.

The electrical conductivity (the inverse of the electrical resistivity) is widely adopted in geophysics to characterise contaminated soil at the field and laboratory scale. The contamination itself, in the case of hydrocarbon compounds, raise the electrical resistivity of the soil, as measured with electrical resistivity tomography (ERT) [43]. The time monitoring of this parameter is widely used to evidence the evolution of contamination, namely its displacement above or below the aquifer or its degradation. In the case of bioremediation, Arato et al. [16] pointed out that while the hydrocarbon contamination increases resistivity values, the biological activity produces ions and cometabolites that may induce a decrease in electrical resistivity. Other Authors [44] investigated the biogeobattery model, according to which the contaminant-degrading microorganisms could induce electrical currents detectable with geophysical surveys as induced polarization (IP). Further studies at the lab scale evidenced electrical conductivity fluctuations in pore water during bioremediation processes [45].

Finally, we want to stress as the application of open-ended coaxial cable on this topic is not yet well diffused. It must be remarked that it does not directly measure electrical conductivity, but a property richer in information: the complex dielectric permittivity, whose imaginary part is linked to conductivity. In such a context, our tests demonstrated how this technology provides detailed information about the dielectric properties of a complex matrix as the soil under biological processes, even if the preliminary tests provided qualitative information. The main limiting factor on the reliability of the open-ended coaxial method in soil studies is the assumption of a homogeneous sample and the quality of the contact with the probe. Therefore, the grain heterogeneities and air bubbles, or roughness in the testing surface can provide for inaccurate measures. We also checked that the accuracy of the device in soil specimen analysis; it appears appropriate for analyzing the behavior at a higher frequency, generally above 2 GHz, but is cold be weak for interpreting the main phenomena related to the low frequencies. This means that the effects caused by fluids (water and hydrocarbons) could be correctly captured as the typical frequency of resonance of saturated or partially saturated soil is above 2 GHz. On the contrary, the interpretation of phenomena related to the biological and chemical effects could be more challenging because those phenomena also interfere with the low-frequency band of the observed spectrum.

## Conclusions

The biostimulation process in two systems with different sizes was compared, after the study of the optimal chemical and physical conditions for diesel oil bioremediation, in terms of water content and carbon to nitrogen ratio [29].

The tested microcosms had the same initial diesel oil concentration (70 g/kg of dry soil), but different amount of soil: 200 g and 800 g of dry soil.

The microcosms with 200 g of dry soil were studied at different water content (u% = 8%, 12% and 15% b.w.) and carbon to nitrogen ratio (C/N = 60, 120, 180 and 300) for 35 days: the microcosm with u% = 12% b.w. and C/N = 180 gave the best results for respirometric and FDA analyses. The microcosms with 800 g of dry soil, tested at u% = 12 b.w. and C/N = 20, 100 and 450, gave the best results for CO_2_ and fluorescein production with C/N = 100.

Moreover, both tests showed that the proper nutrient amount is decisive for the degradation efficiency and very good results can be achieved with water content around 12% by weight and C/N = 100–180.

The difference between the two types of microcosms was the quantity of removed pollutants. For microcosms with 800 g of dry soil, the percentage of removed diesel oil was lower than one of the microcosms with 200 g of dry soil. This result entails that a different geometry or soil amount can influences the biodegradation process, probably as regards oxygen diffusion. This deserves further studies, especially on a larger amount of soil.

The measurements of the complex dielectric permittivity in the microcosms with 200 g of soil were done with an open-ended coaxial cable, a technology not yet used in this context. Findings showed a decrease in the real part, which supports the direct analyses of diesel oil degradation. The variations in the imaginary part, instead, are probably related to changes in the chemistry of pore water; in particular, a nutrient salt depletion was supposed to happen during the test. The approach appears properly working both for observing the permittivity, which could be related to the fluid content and distribution within the soil, and the electrical conductivity, that is more sensitive to biomass activity. The possibility to measure both the parameters in a broad-frequency band will open a new way to monitor biomass activity on soil samples.

## Data Availability

Not applicable.
